# The Hospital's Paying Guest

**Published:** 1923-07

**Authors:** 


THE
### HOSPITAL and HEALTH
REVIEW

### THE HOSPITAL'S PAYING GUEST.

THE extension of hospital benefits to what we may call the middle-class paying guest is rapidly becoming an urgent question. It is a natural development of the contributory system, and a good deal was heard of it at the exceedingly interesting and remarkably well-organised Conference of the Hospitals Association at Sheffield. It came before the Conference in a very definite form in papers read by two distinguished authorities, Sir Napier Burnett and Mr. H. J. Waring, each of whom laid stress upon the change which is taking place in the relation of the Voluntary Hospitals to the public, especially as regards the widening of the areas from which the paying patient is derived. Thus the most novel portion of the valuable and constructive Paper on "Voluntary Hospital Finance," read by Sir Napier Burnett, was devoted to the outlining of a scheme for providing hospital treatment for people with incomes between £250 and £600 or £700 a year. How dire is the case of the professional man of relatively small means who suffers a serious illness—especially if that illness compels surgical assistance—need not be insisted upon. Every doctor, whatever the range of his practice, is painfully aware of it. Wholly or partially, income ceases, heavy additional expenses have to be met, and recovery is retarded by the strain of anxiety. Only too often the patient is ineligible for admission to a general hospital, and is consequently debarred from what Sir Napier Burnett rightly describes as the "wonderful facilities" of the modern hospital. That those facilities should be virtually restricted to the wage-earning classes is an injustice to other *couches sociales*, while the failure to extend them to the community at large can only result in injury to the public health. New medical and surgical knowledge radiates from the hospitals, which are far more than mere institutions for the treatment of disease after it has declared itself, and, to say the least, it is utterly illogical to confine the advantages of that knowledge to the poor and the rich, to the exclusion of that great intermediate class which is the backbone of every country in which it exists.

Sir Napier Burnett looks forward to the time when there shall be a hospital bed available for any member of the community who requires hospital treatment. This may be a counsel of perfection not to be reached until we are all members of the Ideal Republic; but in the meantime it should be obvious that contributory schemes which have been successful for a generation past in the case of the working-classes, ought to be equally successful when they are extended to other classes. The general public has a vague impression that contributory schemes are something new, but when Sir Napier investigated 105 of them he found that the large proportion of 41 have been in existence for more than thirty years, while a further 21 have lasted for between ten and thirty years. We have thus a solid basis of experience upon which to found such extensions as that which this distinguished authority described at Sheffield. Sir Napier Burnett recognises that the first essential is that the actuarial foundations should be sound, but, with that reservation, his proposals are exceedingly simple. For safety's sake he assumes —what is admittedly extravagant—that the percentage of sickness in the middle-class is double the two per cent. of the working class. He further assumes a community of 5,000 people with incomes between £250 and £600 or £700, paying an insurance to the hospital of £2 each per annum. This is roughly ninepence a week, which compares with the penny a week that is the most frequent figure in the existing fixed-amount schemes. The product would be £10,000, the first charge upon which would be the treatment of 200 patients at an average cost to the hospital of £10 each—a total of £2,000. The second charge would be payment to the medical staff to which he would allocate £4,000, which, again for safety's sake, he regards as the maximum. Thus there would remain a sum of at least £4,000, representing "the real insurance quota, or so-called profit" which should be utilised for the provision of increased accommodation and facilities for middle-class patients.

We are in complete agreement with Sir Napier Burnett and Mr. Waring when they argue that hospital medical staffs cannot be expected to give voluntary service to this class of patient. We allow full weight to the fact that these staffs acquire exceedingly valuable, and very varied, experience from their voluntary work in hospitals, but if an appreciable proportion of beds are to be occupied by patients of a different class from that which is usually found in these institutions, we cannot fairly ask the doctors to attend them gratuitously. The voluntary principle does not depend upon free treatment; if it did it would have been abandoned the moment the first contributory scheme came into operation. As to the capacity of the hospitals to accommodate these new patients we need be in little doubt. Sir Napier's 200 patients represent thirteen beds, and a surplus which bore any reasonable relation to his calculations would enable additional accommodation to be provided as it is needed. It may be asked whether the class which Sir Napier Burnett has in view would be prepared to "go into hospital." The simple answer is that a certain number of them go already, with an advantage to themselves which is not adequately shared either by the hospital or the medical staff. Moreover, the hospitals which already treat paying payments have not found it difficult to set aside small wards divided into cubicles. We do not suppose that the author of this scheme is wedded to every one of its details but, broadly speaking, it offers advantages which cannot be disregarded. In addition to a great extension of hospital facilities, it would open up a fresh avenue of finance for the voluntary hospitals, and would provide remuneration for the medical men whose work it would substantially increase. There remains the question of administrative machinery, but that could probably be provided by some extension of the existing contributory scheme organisation. We cannot expect that so novel and far-reaching a plan will get put into execution immediately. Discussion and criticism will be good for it, and some means of organising the middle-classes will have to be found. But it is our duty to the hospitals, our duty, too, to the public health, to press this important subject upon the attention of the country until it becomes a recognised part of our hospital system.

